# Successful treatment of non-small cell lung tumor with 15 lesions by CyberKnife radiosurgery: A case report

**DOI:** 10.3892/etm.2013.1188

**Published:** 2013-06-28

**Authors:** GUIQING YANG, MINGGUANG LI, YISHAN WANG, YUANYUAN WANG, XIAOXU LIU

**Affiliations:** 1 Center for Tumor Treatment, People’s Liberation Army 107th Hospital, Yantai, Shandong 264002, P.R. China; 2 Binzhou Medical College, Lai Shan Qu, Yantai, Shandong 264003, P.R. China

**Keywords:** lung cancer, CyberKnife, stereotactic radiosurgery, case report

## Abstract

Stereotactic body radiation therapy (SBRT) plays an important role in the treatment of early stage non-small cell lung cancer (NSCLC), particularly when patients are unable to tolerate surgical resection due to comorbid conditions or are unwilling to undergo surgery. High rates of local tumor control that may rival the results of surgery have been demonstrated in certain cases with the practical advantage of a short course of treatment and acceptable toxicity. However, there are few reports of a marked change in the complete response of high risk lung cancer with more than ten lesions. In the present study, we report a case of adenocarcinoma of the lung with 15 lesions which had metastasized to the mediastinal lymph nodes. Due to advanced age, multiple lesions and metastasis to the mediastinal lymph nodes and the hilar region of the lung, the patient was treated using CyberKnife. A marked response was noted 42 days after CyberKnife radiosurgery with complete disappearance of the tumor and metastastic lesions.

## Introduction

In 2013, lung cancer was responsible for the majority of cancer deaths at a rate of 37.2/100,000 men (1). Numerous treatment modalities, including surgery, conventional radiotherapy and chemotherapy have been used to provide local control and maximize survival time. When non-small-cell lung cancer (NSCLC) is diagnosed early, for medically suitable patients, the current standard of treatment is surgical resection (2). However, due to comorbid conditions, the majority of patients are considered to be medically inoperable. For these patients who are medically inoperable or unwilling to tolerate surgery, the primary option for treatment is conventional radiotherapy with cumulative doses of ≥60 Gy which are delivered in daily fractions over six or more weeks. However, the five-year survival rate of conventional radiation therapy for these patients is only ~15% and the crude local failure rates is relatively high at 19–70% (3,4). Conventional radiation therapy is only able to improve median overall survival by less than 21 months compared with that for observation alone (5).

With substantial improvements in radiotherapy technology, stereotactic body radiation therapy (SBRT) and dose-intensification has led to promising treatment outcomes. CyberKnife, the robotic stereotactic radiosurgery (SRS) system, which is able to precisely deliver a high dose of radiation, has unique advantages that make it particularly suited for the stereotactic radiotherapy of lung tumors that move with breathing (6). The CyberKnife system sustains accurate and precise radiation delivery by image-guided localization with a compact robotically positioned linear accelerator. The high degree of automated image guidance of CyberKnife enables the successful management of breathing-induced motion and the dynamic tracking of treatment targets without the use of conventional methods (respiratory manipulation and respiratory gating); this is a critical feature that differentiates CyberKnife from other image-guided platforms (7). High tumor control rates in NSCLCs have previously been demonstrated using this novel technique (8). However, the complete response of early non-small cell lung tumors with multiple lesions after CyberKnife therapy has been rarely discussed. We report a case of a stage III lung tumor with 15 lesions that was inoperable due to the advanced age of the patient. The tumor was treated with CyberKnife radiosurgery and a successful response to the new therapy was observed.

## Case report

**Figure 1:**
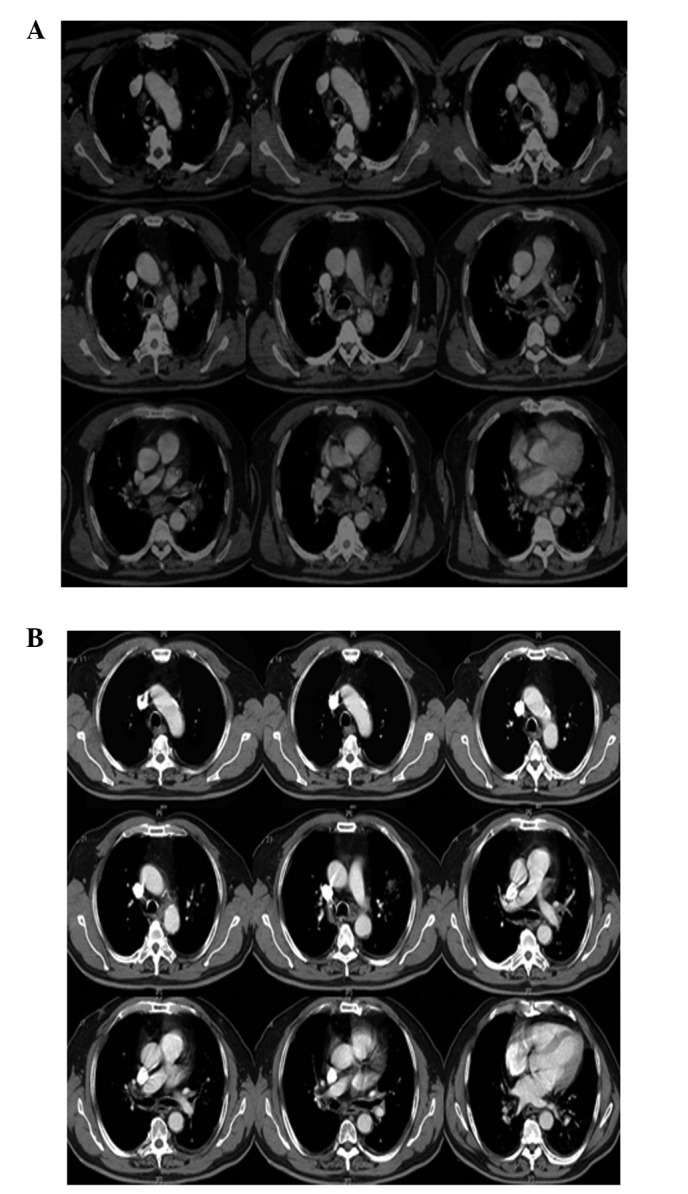
Multiple mediastinal tumors (15 lesions). CT revealed that the tumors disappeared following CyberKnife treatment. (A) CT prior to CyberKnife therapy (January 28, 2011); (B) CT following CyberKnife therapy (March 21, 2011).

A 72-year-old male presented with paroxysmal cough, blood-stained sputum for 1 year and syncope for 5 days prior to hospital admission on January 28, 2011. On admission, a computed tomography (CT) scan demonstrated a 4.4×3.5 cm ring-enhancing lesion on superior posterior segments of the upper lobe of the left lung with a coarse boundary and multiple swollen lymph nodes in the mediastinum as shown in Fig. 1A. The pathology reports confirmed the diagnosis of adenocarcinoma of the lung. Due to the advanced age of the patient, poor body condition and multiple tumor lesions, SRS with CyberKnife was the selected mode of treatment.

The CyberKnife therapy was administered between January 30 and February 17, 2011. The fiducial markers were implanted by conventional bronchoscopy within or adjacent to tumors to serve as targeting references. The two target areas, including 15 lesions, were treated with prescribed doses of 30 and 35 Gy, respectively, to the gross tumor volume (GTV) in 5 fractions. The isodose line covered 85–95% of planning tumor volume (PVT, the precise target of cancer therapy) with 75–85% of the prescription dose. The modified conformity index was 1.41. Clinical examination and CT imaging were performed 42 days after the completion of CyberKnife therapy. A follow-up lung CT scan on March 11, 2011 revealed that no evident lesions were present and the tumor had completely disappeared (Fig. 1B). During routine follow-up, no side-effects due to the radiation were observed. The paroxysmal cough and blood-stained sputum were slowly relieved. CT revealed that tumors had disappeared following CyberKnife treatment (Fig. 1). Written informed consent was obtained from the patient. The study was approved by the Ethics Committee of the People’s Liberation Army 107th Hospital affiliated to Binzhou Medical College (Yantai, China).

## Discussion

Surgical resection is the most effective treatment for patients with early NSCLC. However, for patients who are not able to tolerate surgery, minimally-invasive and effective treatments are required. Among the presently existing radiation treatments, conventional radiotherapy usually leads to severe lung damage due to its low tolerated dose. The total dose of conventional fractionation radiotherapy for early NSCLC is usually 45–66 Gy (9). Dose escalation is one possible strategy for improving the local control rate and enhancing the effect. However, when the dose in 3D conformal radiation therapy (CRT) is increased, the radiation damage of normal tissue is increased. In addition, in order to create a sufficient dosage, the traditional irradiation is prolonged for l0 weeks, which leads to a reduction of local tumor control (10,11).

In 2003, Whyte *et al* treated primary and transfer lung tumors (maximum diameter 1–5 cm) with CyberKnife for the first time and confirmed the feasibility, efficacy and safety of CyberKnife in the treatment of lung cancer (12). The CyberKnife is an image-guided, frameless, real-time robotic radiosurgery system. The benefits of the CyberKnife include more accurate target localization and improved dose delivery for the management of lung tumors. Currently, CyberKnife is widely used for inoperable early lung cancer patients. With a high energy, large dose and narrow beam, CyberKnife is able to focus accurately on the target. CyberKnife treatment has contributed to the optimization of radiotherapy dosing and scheduling, and has improved the efficacy and safety of the radiosurgery treatment. X-rays and γ-rays are able to provide favorable clinical curative effects, but continue to have critical deficiencies due to fixed mounts. By contrast, CyberKnife uses an infrared sensor to track breathing without a frame. CyberKnife is currently the most widely used body SRS technique with controlled synchronous movement of the light beam. Therefore, it is possible to refrain from increasing the irradiation dose due to the mobility of the X-ray beam around the tumor. The equipment also decreases the unnecessary radiation damage to the surrounding normal tissue, which significantly reduces complications. Dynamic target tracking is an integral part of treating tumors that move with respiration using the CyberKnife. This process requires the implantation of metallic fiducial markers in or around the tumor for image-guided tracking. CyberKnife achieves dynamic target tracking using a synchronized respiratory tracking system, and therefore, eliminates the effects of respiratory motion during the treatment process. The implantation of metallic fiducial markers is a key procedure that enables the CyberKnife to realize dynamic target tracking. CT with thin cuts (1.25 mm), which produces high-resolution digitally reconstructed radiographs for optimal position and motion compensation, is used for target delineation and treatment planning (13).

SRS uses multiple convergent beams to deliver a single large dose of radiation to a discrete target volume and is an alternative treatment for patients with advanced lung tumors that are surgically unresectable (8). SRS has been demonstrated to be an effective alternative and noninvasive treatment for advanced lung tumors. Fractionated modality has been considered for the control of larger tumors. The CyberKnife system has been shown to be effective and feasible for use in tackling certain technical challenges involved in these inoperable complex treatments. For smaller, peripheral tumors, high dose radiation therapy (e.g. 60 Gy in 3 fractions) have produced excellent local control results (13). A median 18-month (range, 2–41 months) follow-up study observed that total doses ranging from 5 to 60 Gy delivered in one to four fractions to 35 patients with lung cancer, achieved 71% local control and a survival rate of 77% (14). A multicenter retrospective study analyzed the data of 56 clinical patients treated with CyberKnife. It was observed that the actuarial 2-year local tumor control of patients who received a biologically effective dose >100 Gy was 85%, and the 3-year cancer-specific survival of NSCLC patients was 80% (15). Additional studies are required to optimize the dose for large and centrally located tumors.

In the present case, there was no recurrence of lung tumors following CyberKnife therapy during the follow-up lung-imaging studies. Following CyberKnife radiosurgery with radiation doses of 30 Gy and 35 Gy, the two tumor target areas, which including 15 lesions, were completely resolved within 42 days. During the routine follow-up, no side-effects were observed. In conclusion, the image-guided SRS is particularly useful for lesions it is not possible to treat conventionally, such as lesions that have received the maximal radiation dose using conventional radiotherapy or post-operative residual lesions. The complete response of advanced lung tumors with multiple lesions may also be achieved within a few months after CyberKnife radiosurgery, as shown by the current case study. Despite encouraging preliminary results, longer term follow-up and further clinical trials are required.

## References

[1] Malvezzi M, Bertuccio P, Levi F, La Vecchia C, Negri E (2013). European cancer mortality predictions for the year 2013. Ann Oncol.

[2] Swangsilpa T, Yongvithisatid P, Pairat K, Dechsupa P, Dhanachai M, Dangprasert S, Narkwong L, Sitathanee C, Puataweepong P, Puddhikarant P, Jiarpinitnun C, Witoonpanich P, Ukhumpun T, Khaophong J (2012). Preliminary experience of CyberKnife treatment of primary non-small cell lung cancer. J Med Assoc Thai.

[3] Goldsmith C, Gaya A (2012). Stereotactic ablative body radiotherapy (SABR) for primary and secondary lung tumours. Cancer Imaging.

[4] Qiao X, Tullgren O, Lax I, Sirzén F, Lewensohn R (2003). The role of radiotherapy in treatment of stage I non-small cell lung cancer. Lung Cancer.

[5] Wisnivesky JP, Bonomi M, Henschke C, Iannuzzi M, McGinn T (2005). Radiation therapy for the treatment of unresected stage I-II non-small cell lung cancer. Chest.

[6] Hong JC, Yu Y, Rao AK, Dieterich S, Maxim PG, Le QT, Diehn M, Sze DY, Kothary N, Loo BW (2011). High retention and safety of percutaneously implanted endovascular embolization coils as fiducial markers for image-guided stereotactic ablative radiotherapy of pulmonary tumors. Int J Radiat Oncol Biol Phys.

[7] Brown WT, Wu X, Fayad F, Fowler JF, Amendola BE, García S, Han H, de la Zerda A, Bossart E, Huang Z, Schwade JG (2007). CyberKnife radiosurgery for stage I lung cancer: results at 36 months. Clin Lung Cancer.

[8] Wang YY, Wang YS, Liu T, Yang K, Yang GQ, Liu HC, Wang SS, Yang JL (2013). Efficacy study of CyberKnife stereotactic radiosurgery combined with CIK cell immunotherapy for advanced refractory lung cancer. Exp Ther Med.

[9] Kaskowitz L, Graham MV, Emami B, Halverson KJ, Rush C (1993). Radiation therapy alone for stage I non-small cell lung cancer. Int J Radiat Oncol Biol Phys.

[10] Dosoretz DE, Katin MJ, Blitzer PH, Rubenstein JH, Salenius S, Rashid M, Dosani RA, Mestas G, Siegel AD, Chadha TT (1992). Radiation therapy in the management of medically inoperable carcinoma of the lung: results and implications for future treatment strategies. Int J Radiat Oncol Biol Phys.

[11] Timmerman RD, Kavanagh BD, Cho LC, Papiez L, Xing L (2007). Stereotactic body radiation therapy in multiple organ sites. J Clin Oncol.

[12] Whyte RI, Crownover R, Mushy MJ (2003). Stereotactic radiosurgery for lung tumors: preliminary report of a phase 1 trial. Ann Thorac Surg.

[13] Gibbs IC, Loo BW (2010). CyberKnife stereotactic ablative radiotherapy for lung tumors. Technol Cancer Res Treat.

[14] Brown WT, Wu X, Fowler JF, García S, Fayad F, Amendola BE, de la Zerda A, Schwade JG (2008). Lung metastases treated by CyberKnife image-guided robotic stereotactic radiosurgery at 41 months. South Med J.

[15] Nuyttens JJ, van der Voort van Zyp NC, Praag J, Aluwini S, van Klaveren RJ, Verhoef C, Pattynama PM, Hoogeman MS (2012). Outcome of four-dimensional stereotactic radiotherapy for centrally located lung tumors. Radiother Oncol.

